# Antibacterial and Antivirulence Activities of Acetate, Zinc Oxide Nanoparticles, and Vitamin C Against *E. coli* O157:H7 and *P. aeruginosa*

**DOI:** 10.1007/s00284-022-03151-6

**Published:** 2023-01-02

**Authors:** Selwan Hamed, Mohamed Emara

**Affiliations:** Department of Microbiology and Immunology, Faculty of Pharmacy, Helwan University - Ain Helwan, Helwan, 11795 Egypt

## Abstract

Infectious diseases remain one of the major health challenges worldwide due to the problem of antimicrobial resistance. Conventional antimicrobials have the disadvantage that bacteria rapidly acquire resistance to them, so alternatives must be developed to combat antibiotic resistance. Nanotechnology and the repurposing of existing drugs with known biological profiles are new approaches to replacing conventional antimicrobials. In this paper, we have tested the antibacterial activity of sodium acetate (NaA), vitamin C (VC), and zinc oxide nanoparticles (ZnO NPs) against *Escherichia coli* O157:H7 ATCC 51659 and *Pseudomonas aeruginosa* ATCC 27853*.* MIC values for tested compounds ranged from 0.08 to 6.5 mg ml^−1^, and the effect of combinations and safety profiles against HepG2 cell line of these compounds were also evaluated. At sub-MIC values, tested compounds had a potential antivirulence effect by inhibiting motility and reducing biofilm formation and maturation. Collectively, ZnO NPs and VC are considered safe alternatives to traditional antibiotics that are capable of reducing the development of antibiotic resistance in microbes.

**Graphical Abstract:**

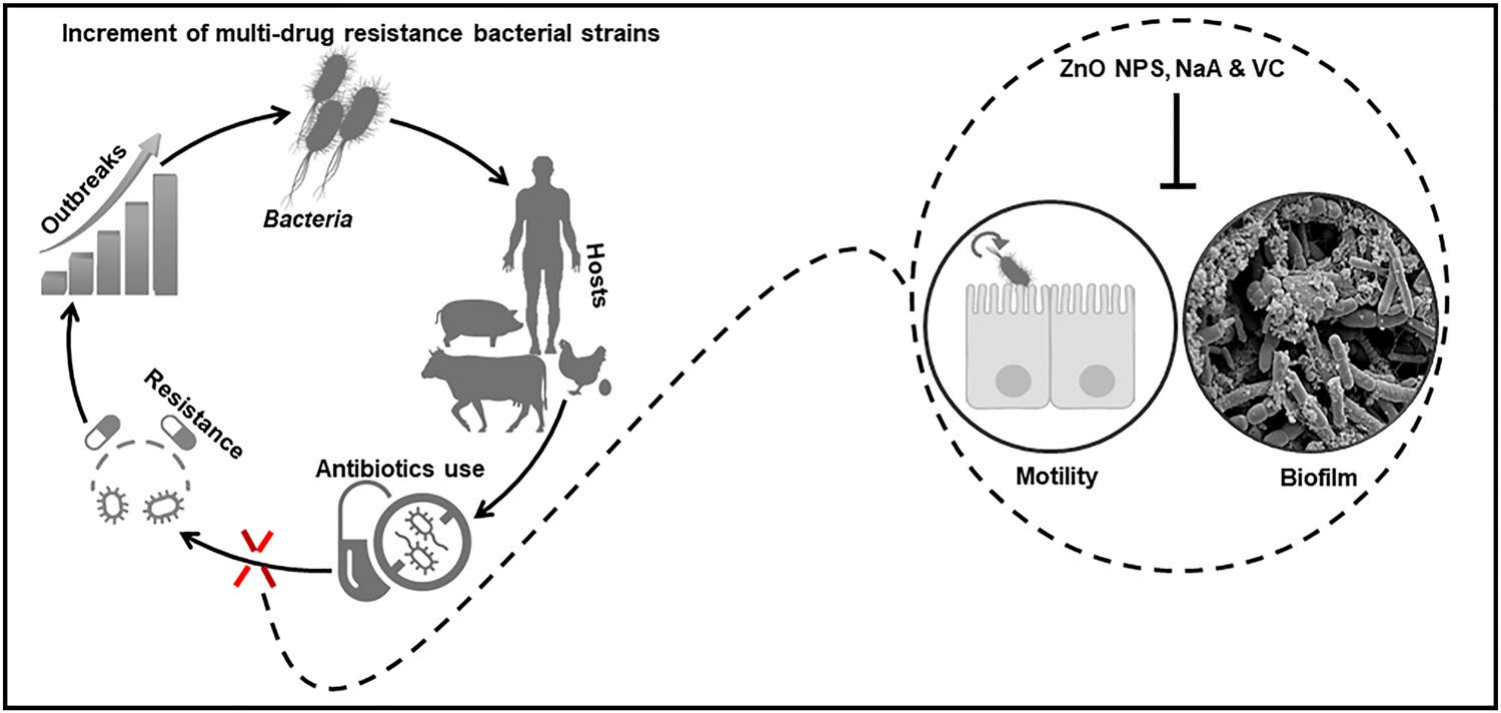

Graphical abstract representing the main aim and the final findings of our work. Spread of multidrug-resistant (MDR) bacterial strains created an urge for alternative safe antimicrobial agents. In this work, we found that ZnO NPs and vitamin C are potential candidates that could be used against MDR *E.coli* and *P. aeruginosa*.

**Supplementary Information:**

The online version contains supplementary material available at 10.1007/s00284-022-03151-6.

## Introduction

The discovery of antibiotics is considered the most significant achievement known to man in the modern era. Despite having a limited spectrum and nature, they contributed to saving many lives during World War II [1]. Unfortunately, bacteria have developed and acquired strategies to resist antibiotics. In turn, this has led to the inevitability of the rapid development of known antibiotics and the development of novel antibiotics that require time, effort, and millions of dollars, creating an additional economic burden [2].

Infections with drug-resistant bacteria have increased morbidity and mortality, hospitalization length, and care costs. Antimicrobial drug resistance is on the rise and is currently responsible for more than 700,000 deaths per year worldwide, according to a report from the World Bank in 2017 [3]. According to WHO reports, it is estimated that 300 million people will die prematurely because of infections with multidrug-resistant organisms over the next 35 years [4, 5]. In addition, the world can expect to lose 60–100 billion US dollars in economic output if antimicrobial drug resistance is not effectively tackled [6].

Therefore, finding different strategies to combat bacterial resistance to antibiotics has become necessary. There are many alternative methods, including nanotechnology, extraction of new antibacterial agents from natural products [7] or the repurposing some safe and FDA-approved drugs or chemicals and testing their efficacy as antibacterial and antivirulence activities [8–10].

One of these alternative methods is nanotechnology [11, 12], in which tiny nanoparticles can target multiple structures in bacteria, making the development of bacterial resistance impossible, Recent studies focused on developing new methods to synthesize effective, safe formulas of nanoparticles either alone or combined with other elements [13, 14]. We are interested in metal and metal oxide nanoparticles as they have superior advantages over other types [15], e.g., reduced size, controlled shape, broad antimicrobial spectrum, and toxicity to multiple bacterial structures; thus, it is challenging for pathogens to develop resistance to metal and metal oxide nanoparticles [16].

On the other hand, repurposing already-approved drugs is another time and effort-saving strategy. Natural and FDA-approved synthetic compounds were tested for their potential antimicrobial and antivirulence activities [17–19]. This approach aims to disarm bacteria by disabling virulence determinants such as motility, adhesion, biofilm formation, and invasion [8, 20].

Gram-negative species are ubiquitous and associated with many infections at different body sites. In addition, they are intrinsically resistant to many antibiotics [21]. Antimicrobial resistance (AMR) in Gram-negative bacteria has become increasingly problematic for healthcare systems over the past years, with escalating costs and mortality [22]. Gram-negative species are frequently associated with nosocomial infections, including bloodstream infections, hospital-acquired pneumonia, urinary tract infections, skin and soft-tissue infections, and complicated intra-abdominal infection [23].

The current work aimed to evaluate the antibacterial and the potential antivirulence activity of ZnO NPs alone or combined with natural substances with known antimicrobial activities, e.g., vitamin C and sodium acetate against *Escherichia coli* O157:H7 ATCC 51659 (*E. coli*) and *Pseudomonas aeruginosa* ATCC 27853 (*P. aeruginosa*).

## Material and Methods

### Bacterial Strains, General Growth Conditions

In this study, *E. coli* and *P. aeruginosa* standard strains were employed, *and E. coli* O157:H7 ATCC 51659 was purchased from Egyptian Microbial Culture Collection (EMCC), Faculty of agriculture, Ain Shams University, while *P. aeruginosa* ATCC 27853 was a gift from Department of Microbiology and Immunology, Faculty of Pharmacy, Tanta University, Egypt. Bacterial strains were grown in Luria–Bertani (LB) at 37° C unless specified elsewhere.

### Reagents

Reagents used in the present study were purchased from Biolab-European Union, Sigma/Aldrich—USA, and Oxoid-UK. All reagents and chemicals used throughout the study were ≥ 99% pure.

### Experimental Design and Statistical Analysis

In this work, we aimed to test the antimicrobial activity of ZnO NPs, VC, and NaA on standard bacterial strains; the potential activity was determined by disk diffusion method for the tested substances versus ofloxacin as a reference standard. MIC for promising substances was determined, then cytotoxicity and antivirulent activity were determined. All experiments were conducted in triplicates. Statistical analysis was performed using OriginPro 2019b (OriginLab, Massachusetts, USA).

### Zinc Oxide Nanoparticles

Zinc oxide nanoparticles (ZnO NPs) used in this study were prepared by wet chemical method as previously described [24]. The prepared nanoparticles were characterized for size and stability using UV–Vis spectrophotometry (T80 + PG instrument) and FEI Tecnai G2 F20 X-TWIN Transmission Electron Microscope (TEM).

### Investigation of the Antibacterial Activity of Tested Compounds

To evaluate the antibacterial activity of sodium acetate (NaA), vitamin C (VC), and ZnO NPs, the standard Kirby-Bauer disk diffusion method was used [25]. Briefly, the overnight cultures of the tested *E. coli* and *P. aeruginosa* strains were diluted to a starting concentration of 1 × 10^8^ CFUml^−1^, and 100 µl of microbial suspensions was spread onto the surface of Muller Hinton agar plates, pH 7.2.

Sterilized filter paper disks with a standard amount of the tested compound (10 µl of 0.82–8.2 mg ml^−1^ of NaA, 20–100 mg ml^−1^ VC and 0.2–1 mg ml^−1^ of ZnO NPs) were then applied to the surface. Plates were then incubated at 37^°^ C overnight. The diameters of the zones of inhibition were measured. Ofloxacin (OFX) was used as reference antibiotic.

### Determination of Minimum Inhibitory Concentrations for the Tested Compounds

The minimum inhibitory concentrations (MICs) of tested compounds were determined using the standard microbroth twofold serial dilution technique following the standard procedures of CLSI [26]. Briefly, overnight cultures of the tested bacterial strains were diluted to a final concentration of 1.5 × 10^5^ CFUml^−1^, and the tested concentrations ranged from 15.6–1, 1.28–8.2, and 0.0156–10 mg ml^−1^ of VC, NaA, and ZnO NPs, respectively. The cultures were then incubated under shaking (~ 150 rpm) at 37° C, and the results were evaluated by measuring the optical density at 600 nm [27]. Ofloxacin (OFX) was used as reference antibiotic. All experiments were conducted in triplicates.

### Evaluating the Effect of the Combination of the Tested Compounds

The combined effect of the VC or NaA with ZnO NPs was evaluated by checkerboard method to obtain the fractional inhibitory concentration (FIC) index [28, 29]. Across the column, each well contained the same concentration of ZnO NPs and then diluted along the x-axis. While in the rows, each well contained the same concentration of VC or SA that was diluted along the y-axis of a 96-well plate.

### Inhibition of Biofilm Formation

In this assay, 10^6^ Cfuml^−1^ of *P. aeruginosa* was incubated in 96-well microtiter plates at subinhibitory concentrations (Sub-MIC) ranging from 0.01 to 0.06 mg ml^−1^ of ZnO NPs, 0.82- 5 mg ml^−1^ of NaA, and 1–6 mg ml^−1^ of VC. Following 24-h incubation at 37^°^ C, planktonic bacteria are rinsed away, and the remaining adherent bacteria (biofilms) are stained with crystal violet dye, thus allowing visualization of the biofilm using absorbance microplate reader (Biotek Elx-808) at 590 nm, the intensity of color is a function of bacterial attachment to the plate and forming a biofilm [30, 31].

Electron microscope imaging (FEI Tecnai G2 F20 X-TWIN Transmission Electron Microscope (TEM)) was used to the study the change in the structure of the formed biofilm. Briefly, a grid/ chip was used to support the formed biofilm that was then washed with phosphate-buffered saline (PBS pH = 7.2); for fixation purpose, the biofilm was treated with 2.5% glutaraldehyde followed by 50, 70, and 100% ethanol and left for air drying.

### Motility Assay

Motility plates were used to quantify the swimming ability of *E. coli* treated with Sub-MIC of tested compounds as specified above in crystal violet assay. Motility plates were prepared using LB broth containing 0.25% of Bacto-agar, sub-MIC of ZnO NPs (0.005:0.06 mg ml^−1^) and (1: 4 mg ml^−1^) of VC were added to each plate, then 1 μl overnight culture of the strain of interest was spotted on to the center of the plate and incubated for 6–8 h. The plates were imaged, and the diameters of the ring formed by motile cells were measured to reflect the swimming ability of cells [32].

### Cytotoxicity Testing

To investigate the cytotoxic effect of tested compounds, HepG2 cells have been employed using the MTT (3-[4,5-dimethylthiazole-2-yl]-2,5- diphenyltetrazolium bromide) cell viability assay, the experiment was conducted at VACSERA Co, Egypt. Briefly, cells (0.5 × 10^5^ cells/ well), in serum-free media, were plated in a flat bottom 96-well microplate and treated with 20 µl of different concentrations of the tested compounds for 48 h at 37º C, in a humidified 5% CO_2_ atmosphere. After incubation, media were removed and 40 µl MTT solution/well was added and incubated again for 4 h, followed by photometric determination of the absorbance at 570 nm using microplate ELISA reader [33, 34]. Experiment was repeated in triplicate.

## Results

As part of our efforts to resolve the issue of antibiotic resistance, we evaluated the antimicrobial and virulence determinants inhibiting the activity of different chemical compounds so that they might be substitutes for conventional antibiotics.

### ZnO NPs, Acetate, and Vitamin C are Promising safe Antibacterial Agents

The prepared ZnO NPs were characterized for size and shape as shown in (Fig.S1). ZnO NPs showed a spherical shape and a size of approximately 50 nM. The disk diffusion technique evaluated the antibacterial potential of ZnO NPs, sodium acetate (NaA), and vitamin C (VC) against standard strains of *E. coli* O157:H7 and *P. aeruginosa.*

The results of Kirby-Bauer disk diffusion illustrated in (Table 1**)** revealed the bactericidal activity of ZnO NPs, NaA, and VC, and the diameters of zones of inhibition were a function of concentration; the diameter increased with increasing the concentration of the tested substance. Zones of inhibition for *E. coli* and *P. aeruginosa* were (15, 17 mm), (23, 27 mm), and (23, 25 mm) at 1, 8.2, and 100 mg ml^−1^ of ZnO NPs, NaA, and VC, respectively.

**Table 1:** Disks diffusion results of ZnO NPs, sodium acetate, and vitamin C alone and in combination against standard *E. coli* and *Pseudomonas aeruginosa*

	OFX	ZnO NPS (mg ml^−1^)			NaA (mg ml^−1^)			VC (mg ml^−1^)		
	0.005 mg per disk	0.2**	0.5*	1*	1.6	4.1**	8.2*	20*	50*	100**
*E. coli*	20*	8*	11**	15*	13*	17**	23**	14*	16*	23**
*P. aeruginosa*	22*	10**	12*	17*	15**	20**	27*	15**	18**	25*

*NaA* sodium acetate and *VC* vitamin C

Diameter of zones of inhibition was measured in mm (The diameter of the used filter paper disk = 6.5 mm)

Results show the mean and standard deviation from three biological replicates, *P < 0.05, **P < 0.001

The broth microdilution technique was used to determine the MICs values for the tested compounds. The results are shown in Table 2. MICs of ZnO NPs, NaA, and VC for *E. coli* and *P. aeruginosa* were (0.1, 0.08 mg ml^−1^), (5- 3.3 mg ml^−1^), and (6–8 mg ml^−1^), respectively.

**Table 2:** MIC values of ZnO NPs, sodium acetate, and vitamin C alone and in combination against standard *E. coli* and *Pseudomonas aeruginosa*

	*E. coli*	*P. aeruginosa*
OFX	0.00006**	0.000045**
ZnO NPs	0.1*	0.08*
NaA	6.5*	5.3**
VC	6**	8*
*Zn/VC	0.015*	0.08**
**Zn/NaA	0.01*	0.06*

*NaA* sodium acetate, *OFX *Ofloxacin

MIC values were determined in mg ml^−1^

Results show the mean and standard deviation from three biological replicates, *P < 0.05, **P < 0.001

We thought to test the effect on MIC values when combining these substances. The effect of the combination of ZnO NPs, NaA, and VC was determined using a checkerboard assay. The fraction inhibitory concentration (FIC) for ZnO NPs at a constant VC concentration of 1 mg ml^−1^ was 0.317 and for ZnO NPs at a constant NaA concentration of 0.82 mg ml^−1^ was 0.27. Interestingly, MIC values of ZnO NPs for *E. coli* and *P. aeruginosa* decreased by 10 and 6.7-folds when combined with 0.82 and 1 mg ml^−1^ of NaA and VC, respectively, as shown in (Table. 2).

**Fig. 1:**
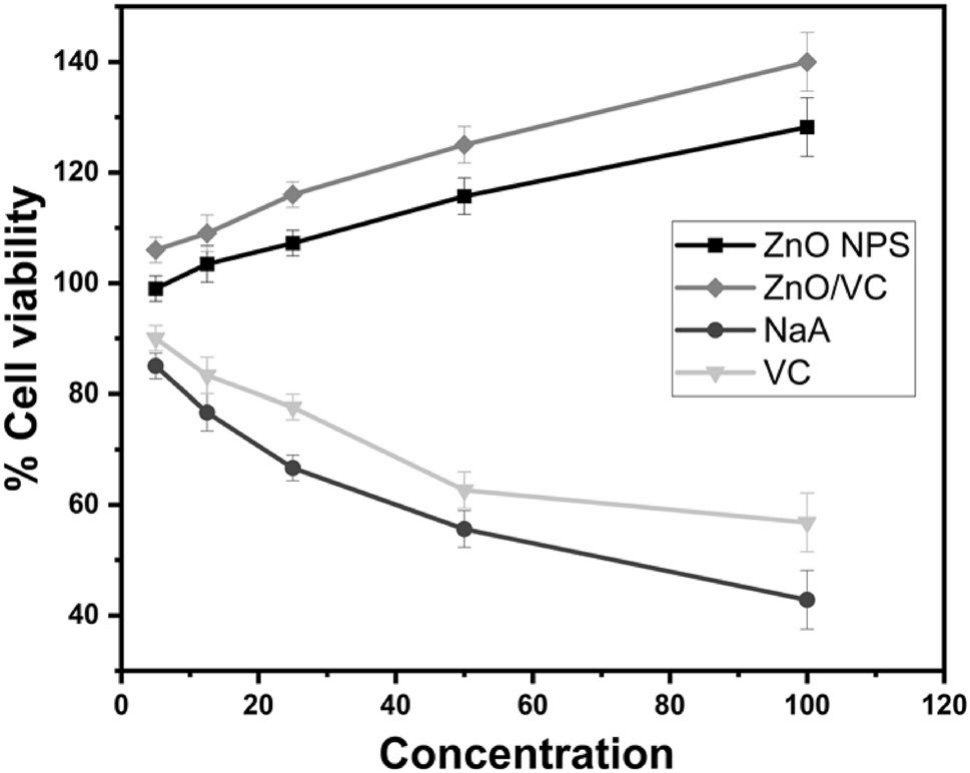
Cytotoxicity of ZnO NPS. ZnO/VC and NaA against HepG2 cell lines. ZnO NPs are safe at MIC value & showed increased cell viability as a function of concentration, besides cell proliferation increased in ZnO/VC combination. In NaA, safety is inversely proportional to concentration, and it showed 70% cell viability around its MIC. Error bars denote the standard deviation from three replicates

ZnO NPs, NaA, and VC were evaluated by MTT assay using HepG2 cell lines, and this showed that a significant increase in cell viability was seen with increasing ZnO NPs and in combined ZnO NPs/VC as shown in (Fig. 1**)**. The viability of cells treated with VC and NaA around the MIC values was 90% for VC and 50–55% for NaA. We did not further study NaA as the viability of cells was low around the MIC. Furthermore, it decreased with an increase in concentration.

### ZnO NPs and Vitamin C Substantially Inhibit Virulence Determinants in Bacteria

**Fig. 2:**
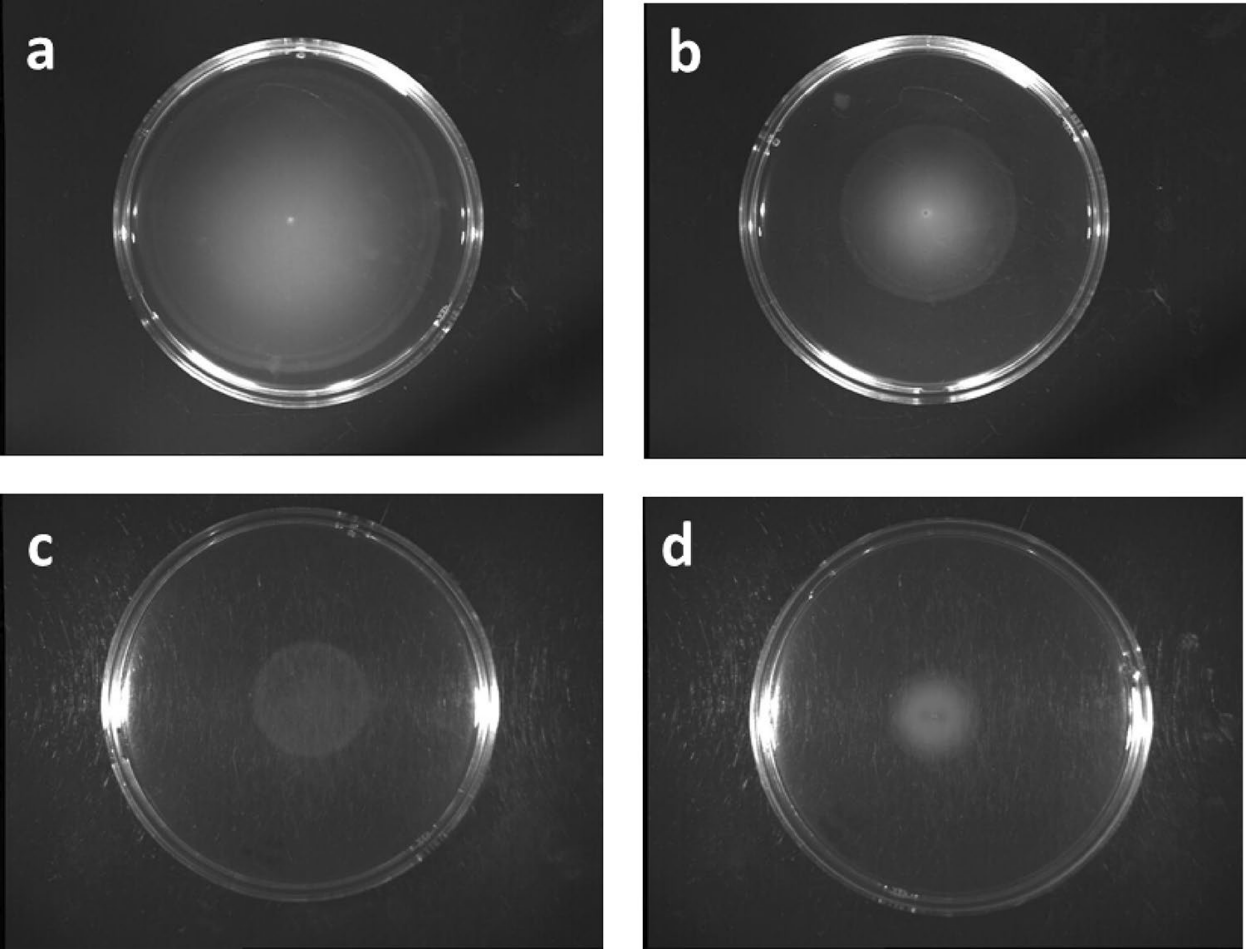
Motility assessment for *E. coli* O157:H7 treated with varying concentrations of ZnO NPs. **a**: negative control (untreated *E. coli*), **b**–**d**: represent increasing concentrations of ZnO NPs (5–30–60 µg ml^−1^), respectively. Motility zone inhibited as a function of increasing the concentration of ZnO NPs. Motility plate assay was conducted in triplicate

We assessed the potential antivirulence activity of ZnO NPs and VC by measuring the inhibition of motility and biofilm formation. A motility plate assay was used where 1 µl of *E. coli* O157:H7 suspension was spotted in the center of the motility plate supplemented with sublethal concentrations of ZnO NPs and VC. The diameters of motility zones decreased by increasing the concentration, as shown in (Fig. 2).

**Fig. 3:**
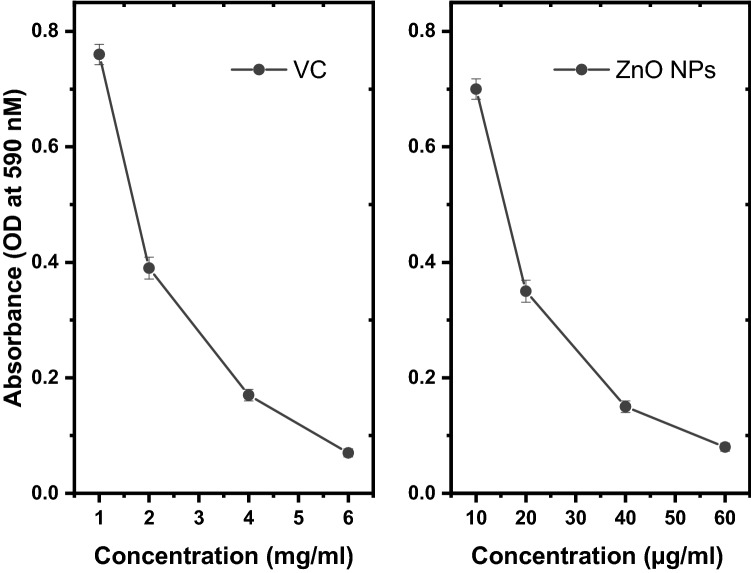
Inhibition of biofilm production of *P. aeruginosa* by ZnO NPs and vitamin C. UV absorption at 590 nm was determined to express the inhibition of biofilm production after treatment of *P. aeruginosa* by a sub-MIC ZnO NPs and VC. Error bars denote the standard deviation from three replicates, *P* < 0.05

To further elucidate the antivirulence activity of ZnO NPs and VC, the effect of tested compounds on biofilm formation was examined. This was done to determine whether biofilms protect against target compounds. Crystal violet microtiter plate assay was used to study biofilm formation by *P. aeruginosa.* This method allows for the observation of bacterial adherence to an abiotic surface. Absorbance at (590 nm) for bacteria treated with Sub-MIC ZnO NPs or VC (Fig. 3) decreased as a function of increasing the tested concentrations.

**Fig. 4:**
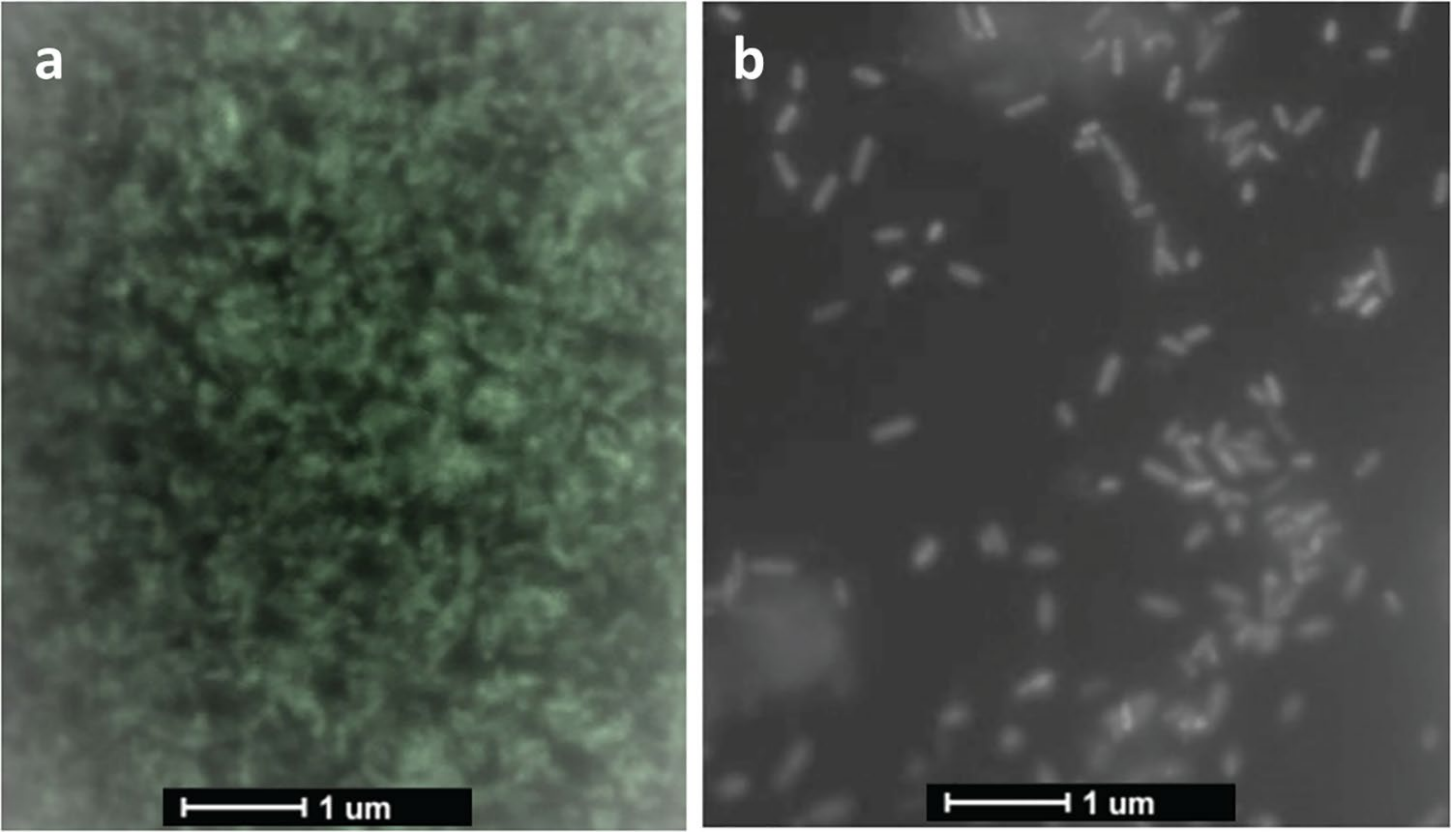
Electron microscope images for *P. aeruginosa* treated with sub-MIC of ZnO NPs and vitamin C. **a**: Untreated bacteria showed cell aggregates and clumps where the biofilm is formed and matured, **b**: after treatment, a loose and less adherent cells are present

Furthermore, electron microscope imaging was used for untreated and treated *P. aeruginosa* at sub-MIC of ZnO NPs to confirm the inhibition of biofilm production (Fig. 4). The intensity of biofilm maturation was inhibited, and planktonic cells were more predominant in treated cells.

## Discussion

The problem of antimicrobial resistance is rapidly becoming a worldwide health crisis. Antimicrobial drugs have been widely used in human medicine for more than 70 years, either as prophylactics or as therapeutics, with tremendous benefits to human health. Unfortunately, the widespread and inappropriate use of antimicrobial drugs has resulted in the emergence of increasing numbers of drug-resistant microbes [35].

If strong measures are not taken to address this most urgent health problem, antimicrobial drug resistance is estimated to cost approximately 10 million lives annually by 2050 worldwide, which is more than cancer and diabetes combined [36].

In this research paper, we aimed to find alternative approaches to combat the problem of antibiotic resistance, specifically in Gram-negative bacteria. We continued our studies on ZnO NPs as potential candidates. We have tested its antimicrobial activity before [37] Here, we evaluated its antivirulence activity alone or combined with other natural substances and chemicals.

Recent studies revealed the antimicrobial and antioxidant activity of vitamin C (VC) and sodium acetate (NaA) [17, 18, 38] against a broad range of bacterial and fungal species in a concentration-dependent manner. This was the impetus to repurpose VC and NaA, evaluate their potential antivirulence activities, determine possible synergism, and their cytotoxic effects at MIC values alone or combined with ZnO NPs against the highly virulent and rapidly developing resistance *E. coli* and *P. aeruginosa* strains.

Disk diffusion and microbroth dilution methods were employed to assess the antibacterial potential of the tested substances, considering the acidity of both VC and NaA. Hence, we measured the activity at pH 4–5. The effect of pH was minor on bacterial growth with VC, but it was drastic with NaA. We fixed the pH in all experiments at pH = 7.2 using buffered media.

Our results support previous studies examining the effect of VC and acetate-containing compounds against Gram-negative bacteria like Klebsiella or carbapenem-resistant Gram-negative strains [19, 39, 40], we tested the antibacterial potential of both VC and NaA against the standard bacterial strains *E. coli* and *P. aeruginosa.* MIC values were (0.1, 0.08 mg ml^−1^), (5–3.3 mg ml^−1^) and (6–8 mg ml^−1^), for ZnO NPs, NaA, and VC for *E. coli* and *P. aeruginosa,* respectively. Interestingly, the tested substances showed synergistic activities when combined as the FIC for the tested substance in combination was < 0.5 [41] and the MIC values of ZnO NPs for *E. coli* and *P. aeruginosa* were reduced by 7–10-folds at fixed concentrations of VC or NaA.

The early detection of the hepatocellular toxicity of newly developed drugs is an essential step during clinical phases of drug approval [42, 43]. Therefore, cytotoxicity of the compounds was evaluated against HepG2 cell lines, and this revealed that both ZnO NPs and ZnO NPs/VC combination were safe around the MIC values where cell viability increased with increasing the concentration. NaA is physiologically present in the small intestine in concentrations of (20–40 mM), and recent studies claimed that NaA acts as a signal to initiate bacterial invasion of small intestine [44, 45]. Increasing the concentrations of NaA above the physiological values could be toxic to the bacteria [46], that’s why NaA was toxic to cells around its MIC values.

Numerous studies showed the regulation and coordination between the expression of virulence determinants and the ability of bacteria to cause disease [44, 47]. Hence, studying the antivirulence effect as well as the antibacterial effect for any promising compounds is necessary. Bacteria can adhere to surfaces and form communities encased in a slime composed of proteins, extracellular polysaccharides, and DNA [40]. Several factors induce biofilm formation including exposure to sublethal concentration of antibiotics.

In addition, biofilms can protect bacteria from antibiotics [48]. Therefore, the antivirulence potential of tested substances were evaluated by testing their ability to inhibit motility of *E. coli* treated with sub-MIC concentrations of ZnO NPs as illustrated in (Fig. 4), similar results were obtained with VC (data not shown). Crystal violet assay and electron microscope imaging were used to examine the antibiofilm activity against *P. aeruginosa*, cell clumps were formed, and biofilm was produced in untreated cells while in the presence of ZnO NPs/VC weekly adherent cells were formed that confirms the ability of the tested substances to inhibit biofilm maturation.

## Conclusion

ZnO NPs, NaA, and vitamin C are safe alternatives to substitute conventional antibiotics. Our results revealed their promising antibacterial and antivirulent activity against the highly virulent *E. coli* O157:H7 and *P. aeruginosa*. Collectively, these results provide potential candidates to solve the problem of resistance to antibiotics. Eventually, we recommend more focused studies on the molecular mechanisms of their action and further *in vivo* evaluation for their safety using animal model.

## Supplementary Information

Below is the link to the electronic supplementary material.

Supplementary file1 (DOCX 361 kb)

## Abbreviations

AMR: Antimicrobial resistance
ATCC: American Type Culture Collection
CFU: Colony forming unit
NaA: Sodium acetate
MDR: Multiple drug resistance
MIC: Minimum inhibitory concentration
OD: Optical density
TEM: Transmission electron microscope
UV–Vis: Ultraviolet- visible
VC: Vitamin C
WHO: World health organization.
ZnO NPs: Zinc oxide nanoparticles
