# Supplementary material for: Antibacterial and Antivirulence Activities of Acetate, Zinc Oxide Nanoparticles, and Vitamin C Against E. coli O157:H7 and P. aeruginosa
Source: Curr Microbiol. 2023 Jan 2;80(2):57. doi: 10.1007/s00284-022-03151-6 (PMC9805986; doi:10.1007/s00284-022-03151-6)
Supplement: Supplementary file 1 — Supplementary file1 (DOCX 361 kb) [file 284_2022_3151_MOESM1_ESM.docx]

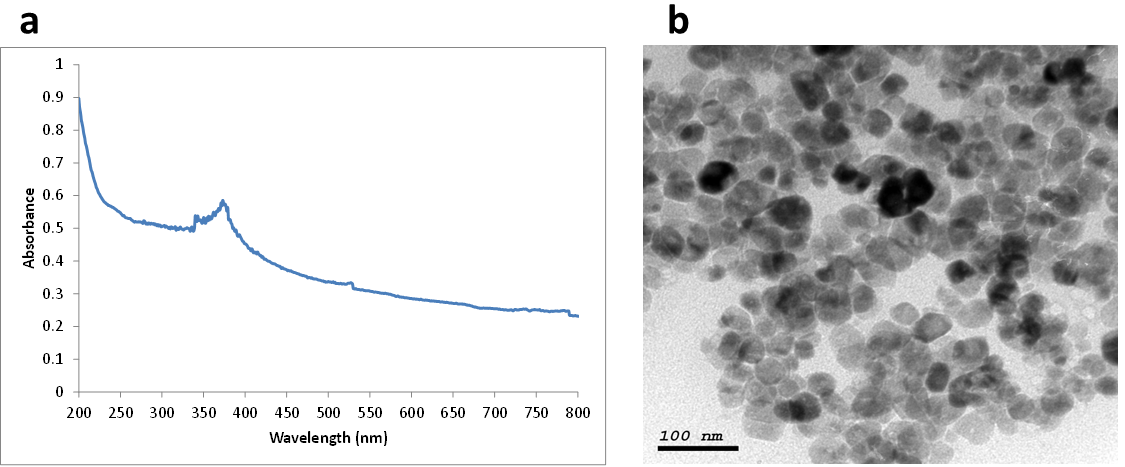


**Fig. S1: Size and shape characterization for the prepared ZnO NPs.** a: UV absorbance spectrum for ZnO NPs, b: TEM image for ZnO NPs revealed average size of 50 nm.
